# Incidence, Prevalence, and Survival of Prostate Cancer in the UK

**DOI:** 10.1001/jamanetworkopen.2024.34622

**Published:** 2024-09-19

**Authors:** Eng Hooi Tan, Edward Burn, Nicola L. Barclay, Antonella Delmestri, Wai Yi Man, Asieh Golozar, Àlvar Roselló Serrano, Talita Duarte-Salles, Philip Cornford, Daniel Prieto Alhambra, Danielle Newby

**Affiliations:** 1 Centre for Statistics in Medicine, Nuffield Department of Orthopaedics, Rheumatology and Musculoskeletal Sciences, University of Oxford, Oxford, United Kingdom; 2 Odysseus Data Service, Cambridge, Massachusetts; 3 Observational Health Data Sciences and Informatics Center at the Roux Institute, Northeastern University, Boston, Massachusetts; 4 Institut Català d’Oncologia, Hospital Universitari Dr Josep Trueta, Girona, Catalonia, Spain; 5 Fundació Institut Universitari per a la recerca a l’Atenció Primària de Salut Jordi Gol i Gurina, Barcelona, Spain; 6 Department of Medical Informatics, Erasmus University Medical Centre, Rotterdam, the Netherlands; 7 Liverpool University Hospitals Foundation Trust, Liverpool, United Kingdom; 8 The University of Liverpool, Liverpool, United Kingdom

## Abstract

**Importance:**

Incidence, prevalence, and survival are pertinent measures to inform the management and provision of prostate cancer care.

**Objective:**

To calculate the incidence, prevalence, and survival rates for prostate cancer in the UK from 2000 to 2021.

**Design, Setting, and Participants:**

This population-based cohort study uses routinely collected primary care data from the UK. Male patients aged 18 years or older with at least 1 year of history registered in Clinical Practice Research Datalink (CPRD) GOLD or Aurum were included. Data were analyzed from January 2023 to March 2024.

**Main Outcomes and Measures:**

Prostate cancer incidence rates (IR), period prevalence (PP), and 1-, 5-, and 10-year survival after diagnosis between 2000 and 2021, stratified by age and calendar years.

**Results:**

This study included 64 925 and 133 200 patients with prostate cancer in CPRD GOLD and Aurum, respectively, with a median age of 72 (65-78) years. The overall IR of prostate cancer was 151.7 (95% CI, 150.6 to 152.9) per 100 000 person-years in GOLD to 153.1 (95% CI, 152.3 to 153.9) per 100 000 person-years for Aurum and increased with age. The incidence of prostate cancer increased from 109 per 100 000 person-years in 2000 to 159 per 100 000 person-years in 2021. Peaks of incidence occurred in 2004 and 2018, before a decline in 2020. PP increased 3.5 times over the study period for both databases, from 0.4% in 2000 to 1.4% in 2021. IR and PP were highest in those aged 80 to 89 years. Median (95% CI) survival was similar in both databases (GOLD: 10.9 [95% CI, 10.7-11.1] years and Aurum: 11.1 [95% CI, 11.0-11.2] years). Survival at 1, 5, and 10 years after diagnosis were 93.4% (95% CI, 93.2%-93.6%), 71.8% (95% CI, 71.4%-72.2%), 53.2% (95% CI, 52.6%-53.7%) in GOLD and 93.9% (95% CI, 93.7%-94.0%), 72.7% (95% CI, 72.5%-73.0%), 53.7% (95% CI, 53.3%-54.1%) in AURUM, respectively. Survival increased over time: 1-year survival was 94.8% (95% CI, 94.5%-95.2%) in those diagnosed between 2015 to 2019 compared with 90.8% (95% CI, 90.2%-91.3%) from 2000 to 2004; 5-year survival improved from 65.3% (95% CI, 64.4%-66.3%) from 2000 to 2004 to 75.3% (95% CI, 74.4%-76.3%) in 2015 to 2019.

**Conclusions and Relevance:**

In this population-based cohort study, incidence and prevalence increased with older age, with high survival rates reflecting a high burden of disease, particularly in the management of cancer survivorship in an aging population. Health care systems should consider this when managing the increasing numbers of people with prevalent prostate cancer.

## Key Points

**Question:**

What is the incidence, prevalence, and survival rates for prostate cancer from 2000 to 2021 using primary care data from the UK?

**Findings:**

In this cohort study of 198 125 patients in 2 UK databases, the incidence of prostate cancer increased from 109 per 100 000 person-years in 2000 to 159 per 100 000 person-years in 2021. Prevalence increased from 0.4% in 2000 to 1.4% in 2021. One-year survival improved from 90.8% in those diagnosed between 2015 to 2019 to 94.8% in 2000 to 2004.

**Meaning:**

These findings suggest that increasing incidence, prevalence, and survival of patients with prostate cancer reflect a high burden in the management of cancer survivorship in an aging population.

## Teaser

This cohort study examines the incidence, prevalence, and survival rates of prostate cancer in the UK from 2000 to 2021.

## Introduction

Prostate cancer (PC) is the most frequently diagnosed cancer and second most common cause of cancer death among men in the United Kingdom (UK).^1^ Population-level screening for PC in asymptomatic men is currently not recommended in the UK as the prostate-specific antigen (PSA) test is neither sensitive nor specific enough for this purpose.^2^ However, healthy men aged over 50 years can ask their general practitioner (GP) for a PSA test.^3^ This is in line with the European Society for Medical Oncology guidelines, which caution universal screening using PSA tests as this could lead to overdiagnosis and overtreatment, with no advantage in overall survival.^4^ Recent awareness campaigns, aimed at identifying and testing those at risk for PC, have been successful in diagnosing cancer at an earlier stage.^5^ With incidence of PC increasing due to an aging population and increased uptake of PSA testing, prevalence is also expected to increase with higher survivorship.

The primary care datasets GOLD and Aurum, contributing to the Clinical Practice Research Datalink (CPRD)^6,7^ in the UK, are collected monthly and provide timely updates to disease surveillance. Although using cancer registry data are ideal to minimize missed diagnosis and inaccurate diagnosis dates compared with primary care data, linkage to cancer registries can be costly^8^ and take more than a year for the data to be accessed. Moreover, coverage periods vary across different cancer registries in England, Scotland, Wales, and Northern Ireland.^9,10,11,12^ Thus, using standardized methods for primary care data spanning various geographic regions in the UK is appealing to describe the trends in disease burden and survival rates, such as PC. This can then be compared with the rates published by the cancer registries. Age-specific and calendar year-specific incidence, prevalence, and survival can help to inform the development of PC management in the UK. The aim of this study was to calculate the incidence, prevalence, and survival rates for PC from 2000 to 2021 using primary care data from the UK, to contribute more evidence on cancer burden and survival trends.

## Methods

### Study Design, Setting, and Data Sources

This population-based cohort study used routinely collected primary care data from the UK. The study protocol was approved by CPRD’s Research Data Governance Process. This report follows the Strengthening the Reporting of Observational Studies in Epidemiology (STROBE) reporting guideline.^13^ Informed consent was not required because patient data were deidentified.

People with a diagnosis of PC and a denominator population were identified from CPRD GOLD to estimate overall survival, incidence, and prevalence. We repeated the analysis in CPRD Aurum because we expected to observe similar trends in the UK. Both databases are broadly representative of the UK population and contain pseudonymized patient-level information about demographics, lifestyle data, clinical diagnoses, prescriptions, and preventive care.^6^ Both databases were mapped to the Observational Medical Outcomes Partnership Common Data Model.^14,15^

### Study Participants

Eligible patients were male, aged 18 years or older, and had at least 1 year of history recorded. For incidence and prevalence analysis, the study cohort consisted of individuals present in the database from January 1, 2000. These individuals were followed up to whichever came first: the cancer outcome of interest, exit from the database, date of death, or the end of study (December 31, 2021, for GOLD, and, due to data availability of latest extraction, December 31, 2019, for Aurum). For the survival analysis, individuals were followed from the date of their cancer diagnosis to either date of death, exit from the database, or end of the study.

### Outcome Definitions

We used diagnostic codes to identify PC. Diagnostic codes indicative of either nonmalignant cancer or metastasis were excluded (apart from prevalence analyses), as well as codes indicative of tumors not originating from prostate tissues. The clinical code list used to define PC was reviewed by clinicians with oncology expertise (eTable 1 in Supplement 1). More than 91% of PC cases in CPRD GOLD were previously confirmed in the cancer registry.^8^ For survival analysis, mortality was defined as all-cause mortality based on date of death records, which have been validated to be more than 98% accurate.^16^

### Statistical Analysis

The characteristics of patients with PC were summarized, with median (IQR) used for continuous variables and counts (percentages) used for categorical variables. For incidence of PC, number of events, observed time at risk, and the IR per 100 000 person-years were summarized with 95% CIs. Overall IR was calculated from 2000 to 2021. Annual IRs were calculated as the number of incident PC cases as the numerator and the person-years in the general population within that year as the denominator. Incident cases were defined as first-ever diagnosis of PC, and they stopped contributing to time at risk after PC diagnosis. Age-standardized IR were calculated using the 2013 European standard population.^17^

Overall and annual period prevalence (PP) was calculated on the first of January for the years 2000 to 2021, with PC cases as the numerator. We included patients with a diagnosis of PC at least once in their observed history, and they continued to contribute to the study after diagnosis. The denominator included individuals in the general population on the first of January in the respective years for each database. The number of events, and prevalence (percentages) were summarized along with 95% CI.

For survival analysis, we used the Kaplan-Meier method^18^ to estimate the overall survival probability with 95% CI. We estimated the median survival and survival probability at 1, 5, and 10 years after diagnosis. Patients whose death and cancer diagnosis occurred on the same date were removed from the survival analysis.

All results were stratified by age. For survival analysis, we additionally stratified by calendar time of cancer diagnosis. To avoid reidentification, we did not report results with fewer than 5 cases. To replicate results from GOLD, the same analysis was performed using CPRD Aurum database, except for stratification by calendar time of cancer diagnosis, which was conducted in GOLD only.

The statistical software R version 4.2.3 (R Project for Statistical Computing) was used for analyses. The analytic code to perform the study is available on Github.^19^ Data were analyzed from January 2023 to March 2024. Data were descriptive and no tests for statistical significance were performed. Two-sided 95% CIs were calculated.

## Results

### Patient Characteristics

Overall, there were 5 539 681 and 11 844 621 eligible male patients 18 years or older, with at least 1 year of history in CPRD GOLD and Aurum, respectively. Attrition tables can be found in eTable 2 in Supplement 1. We included 64 925 patients with PC from the CPRD GOLD database and 133 200 patients with PC from the CPRD Aurum database, with a median (IQR) age of 72 (65-78) years. In both databases, patients with PC had cardiovascular comorbidities, such as heart disease (7710 [11.9%] to 19 443 [14.6%]) and hypertensive disorder (19 094 [29.4%] to 55 406 [41.6%]), as well as osteoarthritis (13 116 [20.2%] to 34 197 [25.7%]), kidney impairment (7712 [11.9%] to 16 508 [12.4%]), and diabetes (6724 [10.4%] to 17 359 [13.0%]) (Table). All study results are available in an interactive web application.^20^

**Table.** Baseline Characteristics of Prostate Cancer Patients at the Time of Diagnosis for Each Database

Database	Patients, No. (%)	
	CPRD GOLD (n = 64 925)	CPRD Aurum (n = 133 200)
Age, median (IQR), y	72 (65-78)	72 (65-78)
Age groups, y		
18-29	6 (<0.1)	10 (<0.1)
30-39	11 (<0.1)	26 (<0.1)
40-49	448 (0.7)	1056 (0.8)
50-59	5841 (9.0)	12 182 (9.1)
60-69	20 168 (31.1)	40 058 (30.1)
70-79	25 025 (38.5)	51 137 (38.4)
80-89	11 916 (18.4)	25 253 (19.0)
≥90	1510 (2.3)	3478 (2.6)
History, median (IQR), d	3632 (1943-5369)	6673 (3333-11 024)
General conditions (any time prior)		
Atrial fibrillation	4448 (6.9)	10 148 (7.6)
Cerebrovascular disease	3867 (6.0)	8890 (6.7)
Chronic liver disease	140 (0.2)	290 (0.2)
Chronic obstructive lung disease	4103 (6.3)	9104 (6.8)
Coronary arteriosclerosis	875 (1.3)	1959 (1.5)
Dementia	647 (1.0)	1509 (1.1)
Depressive disorder	4915 (7.6)	10 417 (7.8)
Diabetes	6724 (10.4)	17 359 (13.0)
Gastroesophageal reflux disease	1707 (2.6)	4106 (3.1)
Gastrointestinal hemorrhage	4871 (7.5)	10 354 (7.8)
Heart failure	1964 (3.0)	4402 (3.3)
Hyperlipidemia	6720 (10.4)	14 929 (11.2)
Hypertensive disorder	19 094 (29.4)	55 406 (41.6)
Ischemic heart disease	7710 (11.9)	19 443 (14.6)
Osteoarthritis	13 116 (20.2)	34 197 (25.7)
Peripheral vascular disease	1170 (1.8)	2669 (2.0)
Pulmonary embolism	641 (1.0)	1741 (1.3)
Kidney impairment	7712 (11.9)	16 508 (12.4)
Venous thrombosis	3017 (4.6)	6690 (5.0)

Abbreviation: CPRD, Clinical Practice Research Datalink.

### Overall and Annual Incidence With Age Stratification

**Figure 1.**
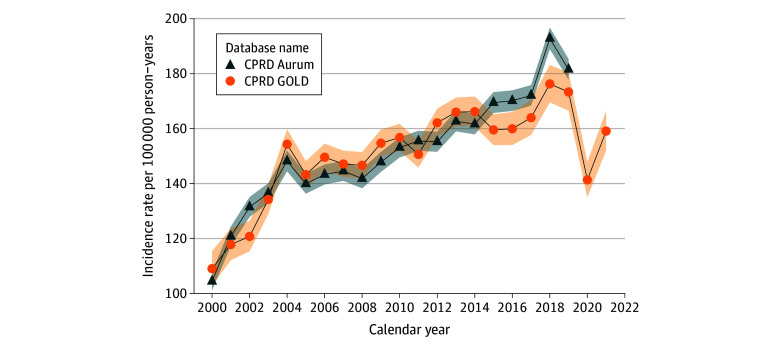
Annualized Incidence Rates of Prostate Cancer Stratified by Database Shaded areas represent 95% CIs. CPRD indicates Clinical Practice Research Datalink.

The overall IR of PC from 2000 to 2021 was 151.7 (95% CI, 150.6-152.9) per 100 000 person-years in GOLD and 153.1 (95% CI, 152.3-153.9) per 100 000 person-years for Aurum. Annualized IRs increased sharply from 2000 to 2004 then gradually until 2018 for both databases. For GOLD, IR decreased in 2020 before increasing again in 2021 but not to levels in 2019 (Figure 1).

Overall IRs were higher with increasing age. Those aged 18 to 29 years had the lowest overall IRs with IRs of 0.07 (95% CI, 0.03-0.16) per 100 000 person-years, whereas those aged 80 to 89 years had the highest IRs of 730.9 (95% CI, 717.8-744.1) per 100 000 person-years for GOLD, with IR of 772.1 (95% CI, 762.6-781.6) per 100 000 person-years for Aurum (eTable 3 in Supplement 1).

**Figure 2.**
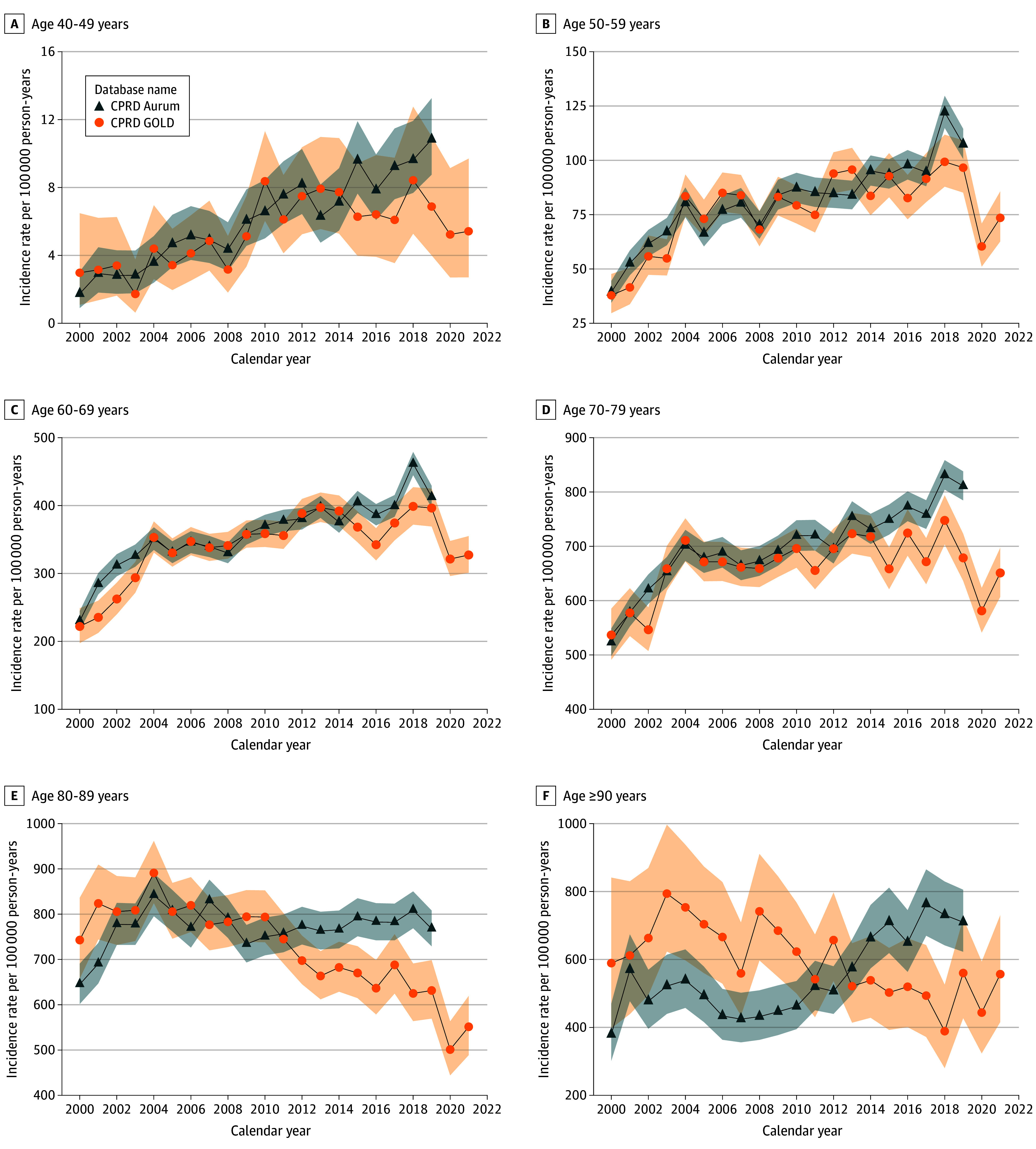
Annualized Incidence Rates of Prostate Cancer Stratified by Database and Age Group Shaded areas represent 95% CIs. There were not enough data points in those aged 18 to 39 years old to assess annualized trends across the study period. CPRD indicates Clinical Practice Research Datalink.

Annualized IRs for each age group (Figure 2) show IRs had steadily increased in those aged 40 to 79 years from during the study for both databases. For those aged 80 years and older in GOLD, there was an overall downward trend in IR over time whereas for Aurum IRs were either stable (80 to 89 years) or increasing (90 years or older). Across all age groups IRs decreased in 2020 before increasing in 2021 in GOLD. Age-standardized IRs were generally higher than crude rates over the years in GOLD (eFigure 1 in Supplement 1).

### Period Prevalence With Age Stratification

In GOLD, PP in 2021 was 1.41% (95% CI, 1.39%-1.43%), and similar between databases in 2019 (1.4%-1.5%). PP increased 3.5 times over the study period for both databases (eFigure 2 in Supplement 1).

**Figure 3.**
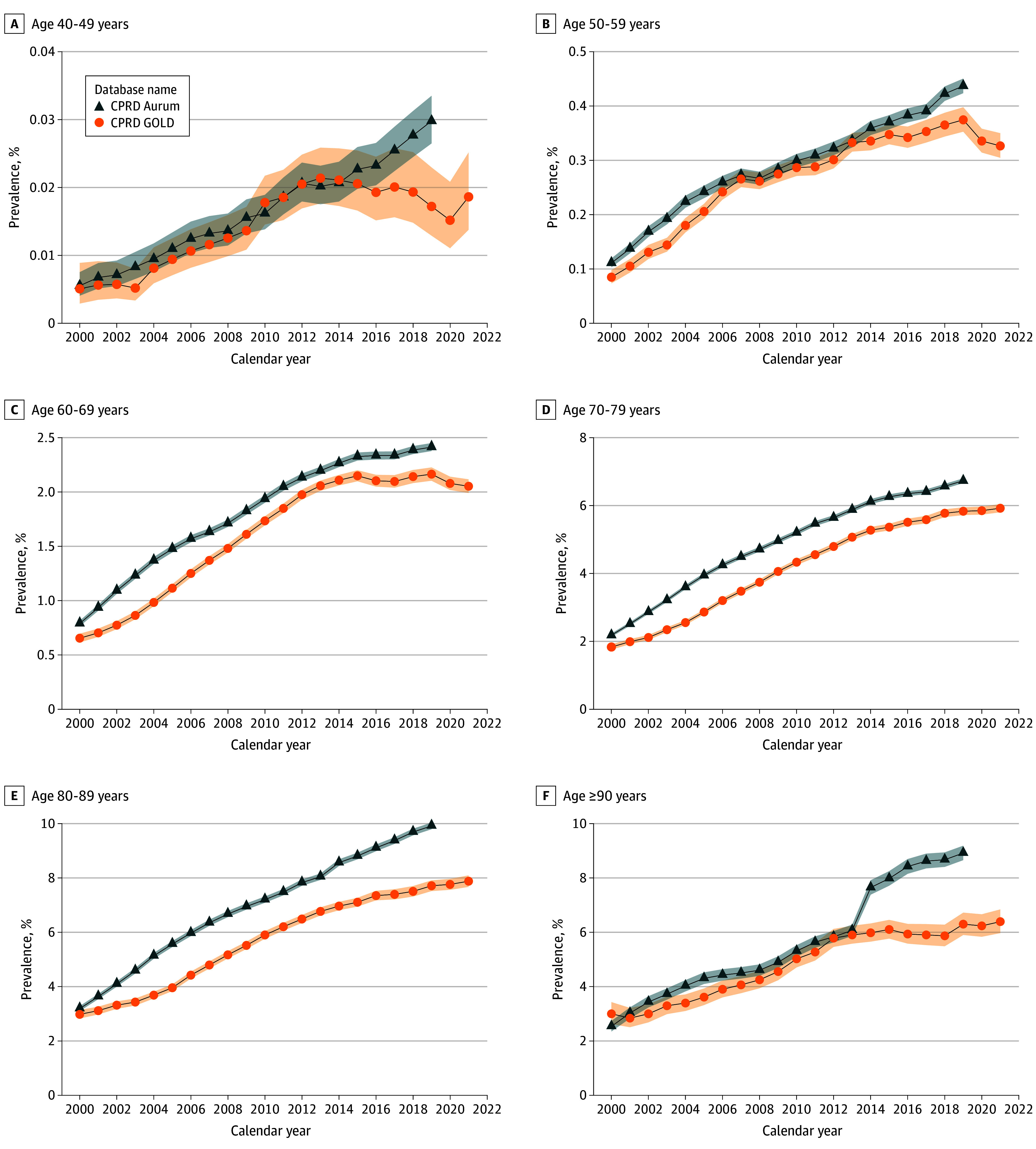
Annualized Period Prevalence of Prostate Cancer Stratified by Database and Age Group Shaded areas represent 95% CIs. There were not enough data points in those aged 18 to 39 years to assess annualized trends. CPRD indicates Clinical Practice Research Datalink.

When stratified by age group, PP in 2021 was highest in 80 to 89 years olds for GOLD (7.7% [95% CI, 7.5%-7.9%]) with similar observations in Aurum in 2019 (9.9% [95% CI, 9.8%-10.0%]). For most age groups, annualized PP increased during the study period with some exceptions (Figure 3). In GOLD, PP in those aged 40 to 49 years increased up until 2014 before stabilizing and declining in 2019 to 2020. There was a drop in PP for those aged 50 to 59 years in 2020 to 2021.

### Survival With Age and Calendar Year Stratification

**Figure 4.**
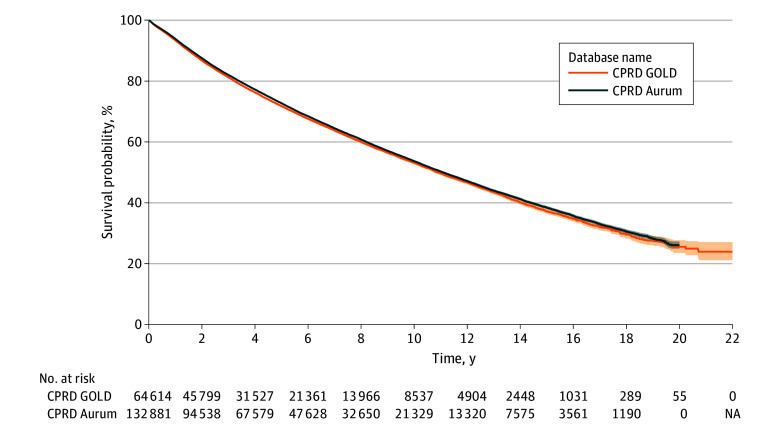
Kaplan-Meier Survival Curve of Prostate Cancer Stratified by Database Shaded areas represent 95% CIs. CPRD indicates Clinical Practice Research Datalink.

In GOLD, there were 64 614 patients with 21 083 deaths over the study period. Median survival in GOLD was 10.9 (95% CI, 10.7-11.1) years, which was similar to the median survival in Aurum (11.1 [95% CI, 11.0-11.2] years) (Figure 4). Survival at 1, 5, and 10 years after diagnosis was 93.4% (95% CI, 93.2%-93.6%), 71.8% (95% CI, 71.4%-72.2%), 53.2% (95% CI, 52.6%-53.7%) for GOLD with similar survival rates in Aurum (eTable 4 in Supplement 1) and numbers at risk reported in eTable 5 in Supplement 1.

Age-stratified median survival was not reached in patients aged between 18 to 59 years and decreased with increasing age across both databases (eTable 6 in Supplement 1). For survival at 1, 5, and 10 years, results were similar between those aged 40 to 49 years as compared with those aged 50 to 59 years, thereafter decreased with increasing age with the lowest survival observed in those aged 90 years and older (eTable 7 in Supplement 1). Results were similar in CPRD Aurum (eTable 8 in Supplement 1).

To investigate if survival has changed over time, we stratified by calendar time of cancer diagnosis in 5-year windows (eFigure 3 in Supplement 1). In CPRD GOLD, improvement in survival was more apparent between the first 3 calendar time windows when PC was diagnosed (2000 to 2004, 2005 to 2009, and 2010 to 2014). Survival was only slightly better in 2015 to 2019 as compared with 2010 to 2014. Survival in those diagnosed during the COVID-19 pandemic in 2020 to 2021 was similar to the previous calendar window. The median survival was not achieved in any years from 2000 to 2021.

For short-term survival, those who were diagnosed between 2015 and 2019 had a higher 1-year survival of 94.8% (95% CI, 94.5%-95.2%) compared with 90.8% (95% CI, 90.2%-91.3%) from 2000 to 2004. Five-year survival improved from 65.3% (95% CI, 64.4%-66.3%) for those diagnosed in 2000 to 2004 to 75.3% (95% CI, 74.4%-76.3%) for those diagnosed between 2015 to 2019 (eTable 9 in Supplement 1). Age-stratified survival was higher in those who were diagnosed between 2015 to 2019 compared with those diagnosed between 2000 to 2004 for those age groups where data was available (40 to 90 years or older) (eTable 10 and 11 in Supplement 1).

## Discussion

This study provides a comprehensive assessment of the trends of PC incidence, prevalence, and survival in the UK from 2000 to 2021. The overall IR of PC in both CPRD GOLD and Aurum databases was 151.7 to 153.1 per 100 000 person-years and increased with age. PP increased 3.5 times throughout the study for both databases from 0.4% in 2000 to 1.4% in 2021. Median survival was 11 years. Survival trends increased over time; 5-year survival improved from 65% in 2000 to 2004 to 75% in 2015 to 2019.

The annual IRs and PPs observed in our study match crude and age-specific rates reported by the National Cancer Registration and Analysis Service (NCRAS).^9^ Due to a migration in software which collates data from GPs, the majority of the contributing practices in CPRD GOLD are now from Scotland (56%) and Wales (28%).^21^ Our study had comparable, albeit higher, crude rates than that reported by the Scottish cancer registry^10^ and lower rates than the Welsh cancer registry.^11^ However, we observed a similar trend of increasing IRs as age increased. We observed a decreasing or stable trend of PC incidence among older men aged 80 to 89 years, which may be reflecting the change of clinical practice to screen for older men with suspicion of PC. UK guidelines^22^ recommended age-based thresholds of PSA levels to refer men for suspected cancer, but this was undefined for men older than 79 years due to lack of evidence, instead clinical judgment should be used.

The increase in PC incidence was mostly attributed to an aging population and the uptake of PSA testing^23^; however, the lack of a formal screening program in UK resulted in variable testing rates across GPs.^24^ We observed 2 peaks of PC incidence in 2004 and 2018. This could be driven by the introduction of the Quality and Outcomes Framework in the UK in 2004, to incentivize GPs with financial rewards for achieving key indicators to improve patient outcomes. Cancer was 1 of the clinical domains monitored, with a register of cancer patients as an indicator.^25^ The peak in 2018 could be attributed to media portrayal of celebrity experience with PC, which led to heightened public awareness and a 36% increase in treatment of urological cancer compared with the previous year.^26^

We observed a sharp decrease in the IR in 2020, especially in patients aged 50 years and older. In the UK, 45% of patients with cancer-related symptoms did not contact their doctor in the immediate months after the COVID-19 pandemic, and cancer referrals fell by 350 000 compared with the previous year.^27^ Our previous analyses demonstrated that PSA testing reduced dramatically during the first 2 years of the COVID-19 pandemic compared with a comparable period before the pandemic, and estimated that between the start of the pandemic and December 2021, around 23% of expected PC diagnoses were missed.^28^ Although we did not observe any difference in short-term survival trends during the COVID-19 pandemic, it will be essential to monitor whether long-term survival is affected.

The 1- and 5-year survival rates in our study closely match those reported by the Office of National Statistics (ONS),^29^ except for the oldest age group aged 90 years or older. It is difficult to disentangle the survival rates as the age stratification in our study was defined differently from the ONS data, where the oldest age stratum is 85 to 99 years. The 5-year survival rates of those aged 80 to 89 years in our study mirror the rates of those aged 85 to 99 years in ONS (46% in 2015 to 2019). In our study, the 5-year survival was only 16% to 23% in those aged 90 years and older. As the life expectancy at age 90 years is around 4 years,^30^ it is reasonable to observe lower survival rates in our study. The number of patients in this age stratum is relatively smaller, so they did not significantly affect the survival rates when grouped together with younger patients. The survival has increased over time, similar to global cancer surveillance trends.^31^ In the UK, 5-year survival rapidly increased from 43% (diagnosed in 1986-1990) to 68% (diagnosed from 1996 to 1999).^32^ In the absence of substantial improvement in treatment of early PC during this time period, this accelerated survival reflected a surge in the diagnosis of men with asymptomatic malignancy, attributed to increased use of PSA tests.^32^ The contribution of PSA testing to reduction in PC mortality is unclear, with contrasting results in randomized clinical trials conducted in Europe and US.^33,34^ Results from a recent trial^35^ showed no difference in mortality between patients with PC assigned to active surveillance vs radical therapy. Instead of radical therapy, active surveillance is recommended to manage low risk patients.^36^ A new trial^37^ to test innovative screening methods such as the use of magnetic resonance imaging in detecting PC was announced in the UK. Improvements in survival in the later calendar period could be attributed to advancements in treatment of metastatic PC. Since the introduction of docetaxel in 2004, doublet and triplet therapies have conferred survival benefit to specific patients with advanced PC.^38^ The overall increase in incidence, prevalence, and survival of PC signals high survivorship which requires preparation of the health care system and adequate funding of necessary services to manage these patients.

The strengths of this study are 3-fold. First, we used 2 large primary care databases covering all of the UK. CPRD Aurum^7^ covers primary care practices in England, whereas CPRD GOLD^6^ covers primary care practices from England, Wales, Scotland, and Northern Ireland. The use of both databases meant it was possible to compare results and increase generalizability of research findings due to greater coverage across the UK. Second, we calculated complete cancer prevalence instead of observed cancer prevalence, which often estimates the number of patients by limiting diagnosis of PC in the past 5 or 10 years.^39^ While many of the patients in our study may be considered cured or not under active treatment after 5 years from diagnosis, with high survival rates in this cancer population, it is necessary to include them in estimating cancer survivorship burden for accurate planning of health care resources. Relying on observed prevalence alone would underestimate the burden of certain subtypes of hematological cancers, particularly those with indolent and curable nature.^39^ Third, we used a well-defined denominator population, which is important in the reproducibility and comparability of cancer IR and PP. A systematic review using population-based cancer registries to calculate colorectal cancer incidence showed only 3% of studies adequately explained the population size estimation procedure to derive IR.^40^

### Limitations

This study has limitations. First, we use primary care data without linkage to the NCRAS data.^41^ Therefore, there is the potential for misclassification and delay in recording of diagnosis leading to selection bias,^8,42^ underestimating incidence and prevalence, and overestimating survival. However, our estimates are in line with national results as previously discussed.

Second, although we did exclude patients who had clinical codes associated with secondary metastatic disease related to PC, we did not exclude patients with a previous history of other cancers before PC diagnosis. Therefore, it is possible that for some patients, cancer in the prostate was not the primary site, which could bias our results and lead to a potential underestimation of survival outcomes. Nonetheless, the number of patients with common cancers, such as colorectal and lung cancer, was low (less than 1%) and would be unlikely to affect the survival estimates.

## Conclusions

In this population-based cohort study, incidence and prevalence increased with older age, with high survival rates reflecting a high burden of disease in the UK, particularly in the management of cancer survivorship in an aging population. Health care systems should consider this to be able to manage the increasing numbers of people with prevalent PC. The use of primary care databases to estimate these trends is helpful for timely assessment of cancer burden. Although we did not observe any difference in short-term survival trends during the COVID-19 pandemic, future studies are needed to examine whether long-term survival is affected.

## Notes

Supplement 1. **eTable 1.** Clinical Code List For Prostate Cancer **eTable 2.** Population Attrition Showing Eligible Patients For Study From Each Database **eTable 3.** Overall Incidence Rates For Prostate Cancer From 2000 to 2021 For GOLD and 2000 to 2019 For Aurum Stratified by Database and Age Group **eFigure 1.** Age-Standardized Incidence Rates For Prostate Cancer From 2000 to 2021 in GOLD **eFigure 2.** Annualized Period Prevalence of Prostate Cancer From 2000 to 2021 for Whole Population **eTable 4.** Survival (%) After 1, 5 and 10 Years After Prostate Cancer Diagnosis Stratified by Database **eTable 5.** Numbers at Risk For Survival For Whole Population and by Age Group For Both CPRD Aurum and GOLD **eTable 6.** Median Survival Stratified by Database and Age Group **eTable 7.** Survival Rates of Prostate Cancer From 2000 to 2021 in CPRD GOLD Stratified by Age Group **eTable 8. **Survival Rates of Prostate Cancer From 2000 to 2019 in CPRD Aurum Stratified by Age Group **eFigure 3.** Kaplan-Meier Survival Curve of Prostate Cancer Stratified by Database and Calendar Year of Diagnosis (2000-2004, 2005-2009, 2010-2014, 2015-2019 and 2020-2021) **eTable 9.** Survival After 1 and 5 Years Stratified by Calendar Year in CPRD GOLD **eTable 10.** Survival After 1 Year Stratified by Age Group and Calendar Year in CPRD GOLD **eTable 11.** Survival After 5 Years Stratified by Age Group, and Calendar Year in CPRD GOLD

Supplement 2.
Data Sharing Statement
