# Supplementary material for: Incidence, Prevalence, and Survival of Prostate Cancer in the UK
Source: JAMA Netw Open. 2024 Sep 19;7(9):e2434622. doi: 10.1001/jamanetworkopen.2024.34622 (PMC12315704; doi:10.1001/jamanetworkopen.2024.34622)
Supplement: Supplement 1. — eTable 1. Clinical Code List For Prostate Cancer eTable 2. Population Attrition Showing Eligible Patients For Study From Each Database eTable 3. Overall Incidence Rates For Prostate Cancer From 2000 to 2021 For GOLD and 2000 to 2019 For Aurum Stratified by Database and Age Group eFigure 1. Age-Standardized Incidence Rates For Prostate Cancer From 2000 to 2021 in GOLD eFigure 2. Annualized Period Prevalence of Prostate Cancer From 2000 to 2021 for Whole Population eTable 4. Survival (%) After 1, 5 and 10 Years After Prostate Cancer Diagnosis Stratified by Database eTable 5. Numbers at Risk For Survival For Whole Population and by Age Group For Both CPRD Aurum and GOLD eTable 6. Median Survival Stratified by Database and Age Group eTable 7. Survival Rates of Prostate Cancer From 2000 to 2021 in CPRD GOLD Stratified by Age Group eTable 8. Survival Rates of Prostate Cancer From 2000 to 2019 in CPRD Aurum Stratified by Age Group eFigure 3. Kaplan-Meier Survival Curve of Prostate Cancer Stratified by Database and Calendar Year of Diagnosis (2000-2004, 2005-2009, 2010-2014, 2015-2019 and 2020-2021) eTable 9. Survival After 1 and 5 Years Stratified by Calendar Year in CPRD GOLD eTable 10. Survival After 1 Year Stratified by Age Group and Calendar Year in CPRD GOLD eTable 11. Survival After 5 Years Stratified by Age Group, and Calendar Year in CPRD GOLD [file jamanetwopen-e2434622-s001.pdf]

Supplemental Online Content

Tan EH, Burn E, Barclay NL, etc. Incidence, prevalence, and survival of prostate cancer in the UK. *JAMA Netw Open*. 2024;7(9):e2434622. doi:10.1001/jamanetworkopen.2024.34622

eTable 1. Clinical Code List For Prostate Cancer

eTable 2. Population Attrition Showing Eligible Patients For Study From Each Database

eTable 3. Overall Incidence Rates For Prostate Cancer From 2000 to 2021 For GOLD and 2000 to 2019 For Aurum Stratified by Database and Age Group

eFigure 1. Age-Standardized Incidence Rates For Prostate Cancer From 2000 to 2021 in GOLD

eFigure 2. Annualized Period Prevalence of Prostate Cancer From 2000 to 2021 for Whole Population

eTable 4. Survival (%) After 1, 5 and 10 Years After Prostate Cancer Diagnosis Stratified by Database

eTable 5. Numbers at Risk For Survival For Whole Population and by Age Group For Both CPRD Aurum and GOLD

eTable 6. Median Survival Stratified by Database and Age Group

eTable 7. Survival Rates of Prostate Cancer From 2000 to 2021 in CPRD GOLD Stratified by Age Group

eTable 8. Survival Rates of Prostate Cancer From 2000 to 2019 in CPRD Aurum Stratified by Age Group

eFigure 3. Kaplan-Meier Survival Curve of Prostate Cancer Stratified by Database and Calendar Year of Diagnosis (2000-2004, 2005-2009, 2010-2014, 2015-2019 and 2020-2021)

eTable 9. Survival After 1 and 5 Years Stratified by Calendar Year in CPRD GOLD

eTable 10. Survival After 1 Year Stratified by Age Group and Calendar Year in CPRD GOLD

eTable 11. Survival After 5 Years Stratified by Age Group, and Calendar Year in CPRD GOLD

This supplemental material has been provided by the authors to give readers additional information about their work.

© 2024 Tan EH et al. *JAMA Network Open*.

eTable 1: Clinical Code List For Prostate Cancer The clinical codelist used for prostate cancer is listed in the table below with the corresponding SNOMED concept ID, OMOP concept ID and concept description. Only diagnosis records alone were used to identify cancer outcome for this study. Different codelists were created for incident and prevalent definitions of prostate cancer. We developed concept definitions using ATLAS, the OHDSI open-source platform (https://github.com/OHDSI/atlas). Clinical adjudicators reviewed the cohort definitions and associated concept sets. Additionally, a detailed description of the definition used to identify the prostate cancer outcome for this study is provided at an interactive web application (https://dpa-pde-oxford.shinyapps.io/EHDENCancerIncPrevCohortDiagShiny/).

Concept Id	Concept SNOMED Code	Concept Description	Code used for
4163261	399068003	Malignant tumor of prostate	Incidence and prevalence
200962	93974005	Primary malignant neoplasm of prostate	Incidence and prevalence
4082919	278060005	Endometrioid carcinoma of prostate	Incidence and prevalence
4116087	254900004	Carcinoma of prostate	Incidence and prevalence
4141960	427492003	Hormone refractory prostate cancer	Incidence and prevalence
4161028	399490008	Adenocarcinoma of prostate	Incidence and prevalence
4164017	399590005	Squamous cell carcinoma of prostate	Incidence and prevalence
4288534	396198006	Small cell carcinoma of prostate	Incidence and prevalence
36684947	459381000124106	Metastatic castration-resistant prostate cancer	Incidence and prevalence
36716186	722103009	Hormone sensitive prostate cancer	Incidence and prevalence
37311236	823017009	Infiltrating duct carcinoma of prostate	Incidence and prevalence
37311683	822970008	Acinar cell cystadenocarcinoma of prostate	Incidence and prevalence
37395835	715412008	Familial prostate cancer	Incidence and prevalence
764971	96901000119105	Prostate cancer metastatic to eye	Prevalence only
4196262	314994000	Metastasis from malignant tumor of prostate	Prevalence only
4200890	314969001	Local recurrence of malignant tumor of prostate	Prevalence only
36712762	1098981000119100	Recurrent malignant neoplasm of prostate	Prevalence only
37016740	712849003	Prostate cancer metastatic to bone	Prevalence only

© 2024 Tan EH et al. *JAMA Network Open*.

eTable 2: Population Attrition Showing Eligible Patients For Study From Each Database

N	Reason	N excluded	Database
39999011	Starting population		Aurum
39999011	Missing year of birth	0	
39999011	Missing sex	0	
34833388	Cannot satisfy age criteria during the study period based on year of birth	5165623	
29190480	No observation time available during study period	5642908	
29190480	Doesn't satisfy age criteria during the study period	0	
25483313	Prior history requirement not fulfilled during study period	3707167	
12425473	Not Male	13057840	
11844621	No observation time available after applying age and prior history criteria	580852	
11844621	Starting analysis population	0	
11844621	Estimating prevalence	0	
11817433	Excluded due to prior event (do not pass outcome washout during study period)	27188	
11817433	Estimating incidence	11684233	
133200	With a cancer diagnosis	319	
132881	Cancer diagnosis not on same date as death	0	
132881	Estimating survival		
17054819	Starting population		GOLD
17054819	Missing year of birth	0	
17054819	Missing sex	0	
15210165	Cannot satisfy age criteria during the study period based on year of birth	1844654	
13978229	No observation time available during study period	1231936	
13978229	Doesn't satisfy age criteria during the study period	0	
12254874	Prior history requirement not fulfilled during study period	1723355	
5979681	Not Male	6275193	
5539681	No observation time available after applying age and prior history criteria	440000	
5539681	Starting analysis population	0	
5539681	Estimating prevalence	0	
5531493	Excluded due to prior event (do not pass outcome washout during study period)	8188	
5531493	Estimating incidence	0	
64925	With a cancer diagnosis	5466568	
64614	Cancer diagnosis not on same date as death	311	
64614	Estimating survival		

© 2024 Tan EH et al. *JAMA Network Open*.

eTable3: Overall Incidence Rates For Prostate Cancer From 2000 to 2021 For GOLD and 2000 to 2019 For Aurum Stratified by Database and Age Group

Database	Age Group (years)	n persons	person years	n events	Incidence (per 100,000 pys)
Aurum	18 to 29	4,336,626	16,551,873	10	0.06 (0.03 to 0.11)
	30 to 39	4,204,748	16,690,760	26	0.16 (0.10 to 0.23)
	40 to 49	3,420,860	16,954,009	1,056	6.23 (5.86 to 6.62)
	50 to 59	2,795,244	14,642,516	12,182	83.20 (81.73 to 84.69)
	60 to 69	2,083,887	11,025,017	40,058	363.34 (359.79 to 366.91)
	70 to 79	1,431,674	7,233,343	51,137	706.96 (700.85 to 713.12)
	80 to 89	748,744	3,270,891	25,253	772.05 (762.56 to 781.63)
	90 +	190,782	630,170	3,478	551.91 (533.72 to 570.57)
GOLD	18 to 29	1,836,495	8,110,895	6	0.07 (0.03 to 0.16)
	30 to 39	1,861,599	7,627,705	11	0.14 (0.07 to 0.26)
	40 to 49	1,680,234	8,203,703	448	5.46 (4.97 to 5.99)
	50 to 59	1,468,804	7,426,710	5,841	78.65 (76.64 to 80.69)
	60 to 69	1,137,655	5,792,587	20,168	348.17 (343.38 to 353.01)
	70 to 79	771,134	3,740,073	25,025	669.10 (660.84 to 677.45)
	80 to 89	392,472	1,630,410	11,916	730.86 (717.79 to 744.10)
	90 +	90,708	254,824	1,511	592.96 (563.43 to 623.63)

pys: person years

© 2024 Tan EH et al. *JAMA Network Open*.

eFigure 1: Age-Standardized Incidence Rates For Prostate Cancer From 2000 to 2021 in GOLD

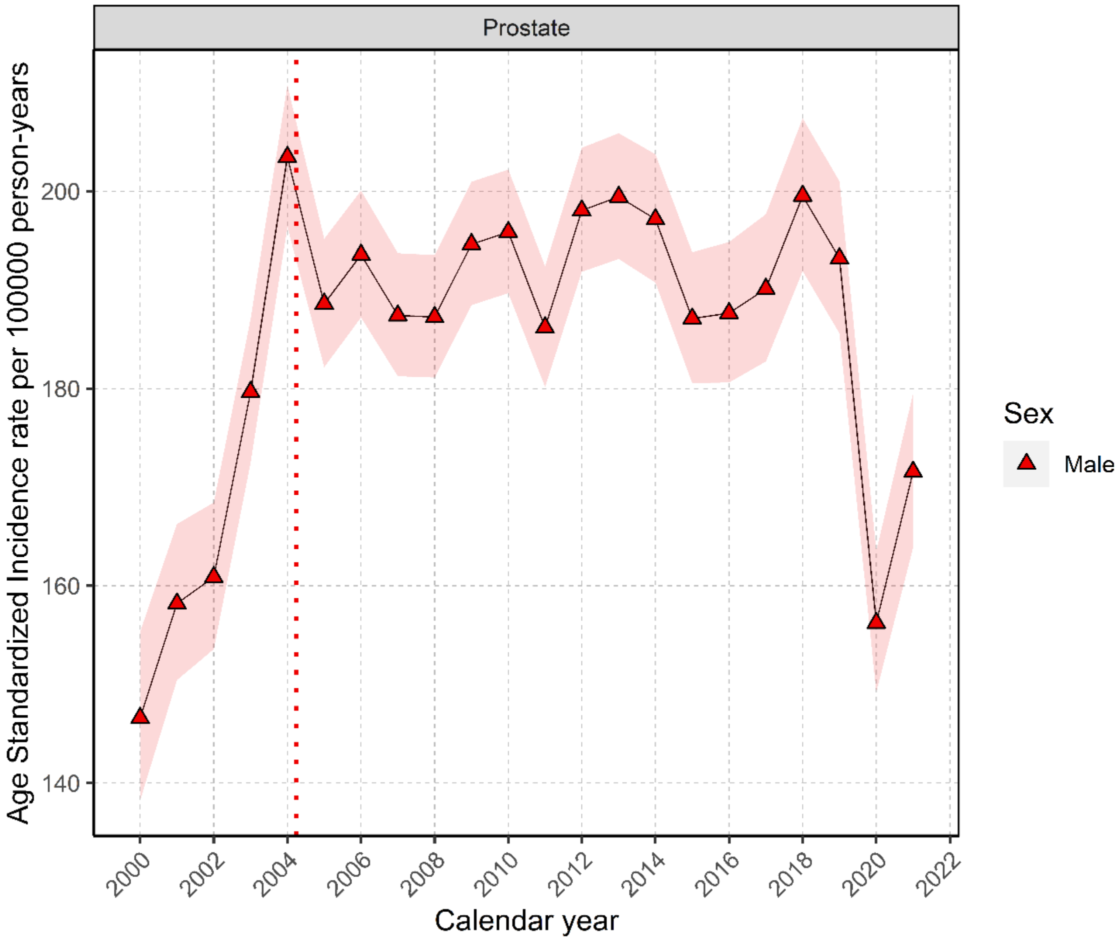

Note: standardised to the European Standard Population 2013

© 2024 Tan EH et al. *JAMA Network Open*.

eFigure 2: Annualized Period Prevalence of Prostate Cancer From 2000 to 2021 for Whole Population

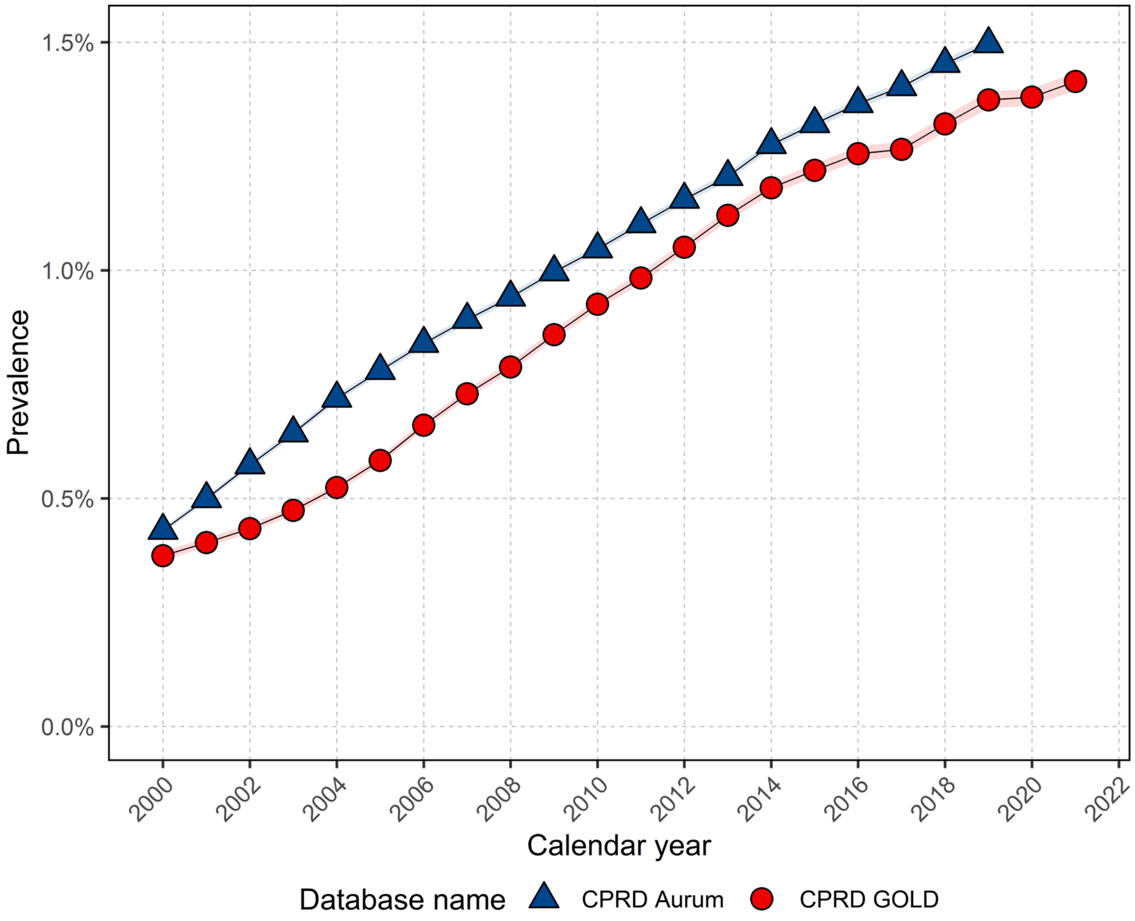

eTable 4: Survival (%) After 1, 5 and 10 Years After Prostate Cancer Diagnosis Stratified by Database

Database	Time	% Survival (95% CI)
Aurum	1	93.9 (93.7 to 94.0)
	5	72.7 (72.5 to 73.0)
	10	53.7 (53.3 to 54.1)
GOLD	1	93.4 (93.2 to 93.6)
	5	71.8 (71.4 to 72.2)
	10	53.2 (52.6 to 53.7)

CI confidence interval

© 2024 Tan EH et al. *JAMA Network Open*.

eTable 5: Numbers at Risk For Survival For Whole Population and by Age Group For Both CPRD Aurum and GOLD

Time (Years)	Details	0	2	4	6	8	10	12	14	16	18	20	Database
All	n risk	132881	94538	67579	47628	32650	21329	13320	7575	3561	1190	-	CPRD Aurum
	n event	15014	10102	6900	4721	3329	2213	1388	786	361	88	-	
18 to 29	n risk	10	<5	<5	0	0	0	0	0	0	0	-	
	n event	<5	<5	<5	0	0	0	0	0	0	0	-	
30 to 39	n risk	26	17	11	7	5	<5	<5	<5	0	0	-	
	n event	<5	<5	<5	<5	<5	<5	<5	<5	0	0	-	
40 to 49	n risk	1056	775	578	419	303	200	127	77	40	16	-	
	n event	42	31	17	7	<5	9	<5	<5	<5	<5	-	
50 to 59	n risk	12173	9226	7052	5276	3981	2833	1993	1272	666	247	-	
	n event	422	351	262	148	144	97	64	50	25	6	-	
60 to 69	n risk	40025	31108	23984	18199	13355	9379	6243	3779	1882	657	-	
	n event	2036	1701	1250	966	748	585	409	282	150	46	-	
70 to 79	n risk	51044	37179	26703	18733	12533	7830	4543	2311	934	263	-	
	n event	5126	3960	2973	2199	1690	1156	776	401	175	33	-	
80 to 89	n risk	25117	14982	8764	4832	2421	1071	409	133	38	7	-	
	n event	5922	3537	2190	1331	720	357	135	52	11	<5	-	
90 +	n risk	3430	1247	486	162	52	12	<5	0	0	0	-	
	n event	1463	521	208	70	23	9	<5	0	0	0	-	
All	n risk	64614	45799	31527	21361	13966	8537	4904	2448	1031	289	55	CPRD GOLD
	n event	7778	4955	3159	2081	1348	865	533	243	97	22	<5	
18 to 29	n risk	6	<5	<5	<5	<5	0	0	0	0	0	0	
	n event	<5	<5	<5	<5	<5	0	0	0	0	0	0	
30 to 39	n risk	11	8	7	<5	<5	<5	<5	0	0	0	0	
	n event	<5	<5	<5	<5	<5	<5	<5	0	0	0	0	
40 to 49	n risk	448	346	257	183	138	83	42	23	10	6	<5	

© 2024 Tan EH et al. *JAMA Network Open*.

	n event	22	13	<5	<5	<5	<5	<5	<5	<5	<5	<5
50 to 59	n risk	5836	4588	3357	2466	1792	1176	762	472	224	80	17
	n event	213	194	101	57	52	41	28	11	6	<5	<5
60 to 69	n risk	20143	15436	11425	8309	5745	3805	2331	1213	535	141	30
	n event	1153	895	636	451	307	230	175	86	41	12	<5
70 to 79	n risk	24949	17978	12388	8275	5256	3011	1622	692	255	60	6
	n event	2796	1983	1400	1001	694	457	287	128	50	7	<5
80 to 89	n risk	11771	6894	3893	2049	1014	454	143	45	5	<5	<5
	n event	2970	1640	946	536	283	133	42	18	<5	<5	<5
90 +	n risk	1450	543	196	72	15	<5	0	0	0	0	0
	n event	624	229	73	32	9	<5	0	0	0	0	0

© 2024 Tan EH et al. *JAMA Network Open*.

eTable 6: Median Survival Stratified by Database and Age Group

Database	Age Group	Median survival (years)	n persons	n events
Aurum	60 to 69	18.05 (17.71 to 18.43)	40025	8173
	70 to 79	10.09 (9.95 to 10.24)	51044	18489
	80 to 89	4.58 (4.49 to 4.67)	25117	14258
	90 +	2.08 (1.97 to 2.19)	3430	2296
GOLD	60 to 69	17.41 (16.64 to 18.13)	20143	3987
	70 to 79	9.68 (9.47 to 9.914)	24949	8804
	80 to 89	4.32 (4.22 to 4.43)	11771	6568
	90 +	2.08 (1.90 to 2.36)	1450	969

For ages 18-39 years median survival and confidence interval results obscured due to small cell counts and ages 40-59 years median survival not achieved

eTable 7: Survival Rates of Prostate Cancer From 2000 to 2021 in CPRD GOLD Stratified by Age Group

Age Group (years)	One year survival (%)	Five-year survival (%)	Ten-year survival (%)
18-29	100	-	-
30-39	100	-	-
40-49	96.9 (95.2 to 98.6)	89.2 (86.0 to 92.6)	84.4 (79.7 to 89.3)
50-59	98.1 (97.7 to 98.5)	89.7 (88.8 to 90.6)	82.9 (81.5 to 84.2)
60-69	97.3 (97.1 to 97.5)	84.9 (84.4 to 85.5)	72.1 (71.2 to 73.0)
70-79	94.2 (93.9 to 94.5)	72.0 (71.3 to 72.6)	48.7 (47.8 to 49.7)
80-89	85.3 (84.6 to 86.0)	44.7 (43.7 to 45.8)	17.8 (16.7 to 18.9)
90+	70.8 (68.4 to 73.3)	19.4 (16.9 to 22.3)	1.6 (0.5 to 4.8)

eTable 8: Survival Rates of Prostate Cancer From 2000 to 2019 in CPRD Aurum Stratified by Age Group

Age Group	One year survival (%)	Five-year survival (%)	Ten-year survival (%)
18-29	-	-	-
30-39	100	84.2 (69.3 to 100)	-
40-49	98.6 (97.8 to 99.3)	89.4 (87.2 to 91.6)	84.9 (82.0 to 87.9)
50-59	98.5 (98.2 to 98.7)	89.9 (89.3 to 90.6)	81.9 (80.9 to 82.8)
60-69	97.6 (97.5 to 97.8)	86.1 (85.7 to 86.5)	73.5 (73.0 to 74.1)
70-79	95.0 (94.8 to 95.2)	73.6 (73.2 to 74.0)	50.4 (49.8 to 51.0)
80-89	86.4 (86.0 to 86.9)	46.7 (45.9 to 47.4)	17.5 (16.7 to 18.2)
90+	69.7 (68.1 to 71.4)	19.2 (17.6 to 21.0)	2.7 (1.8 to 4.2)

© 2024 Tan EH et al. *JAMA Network Open*.

eFigure 3: Kaplan-Meier Survival Curve of Prostate Cancer Stratified by Database and Calendar Year of Diagnosis (2000-2004, 2005-2009, 2010-2014, 2015-2019 and 2020-2021)

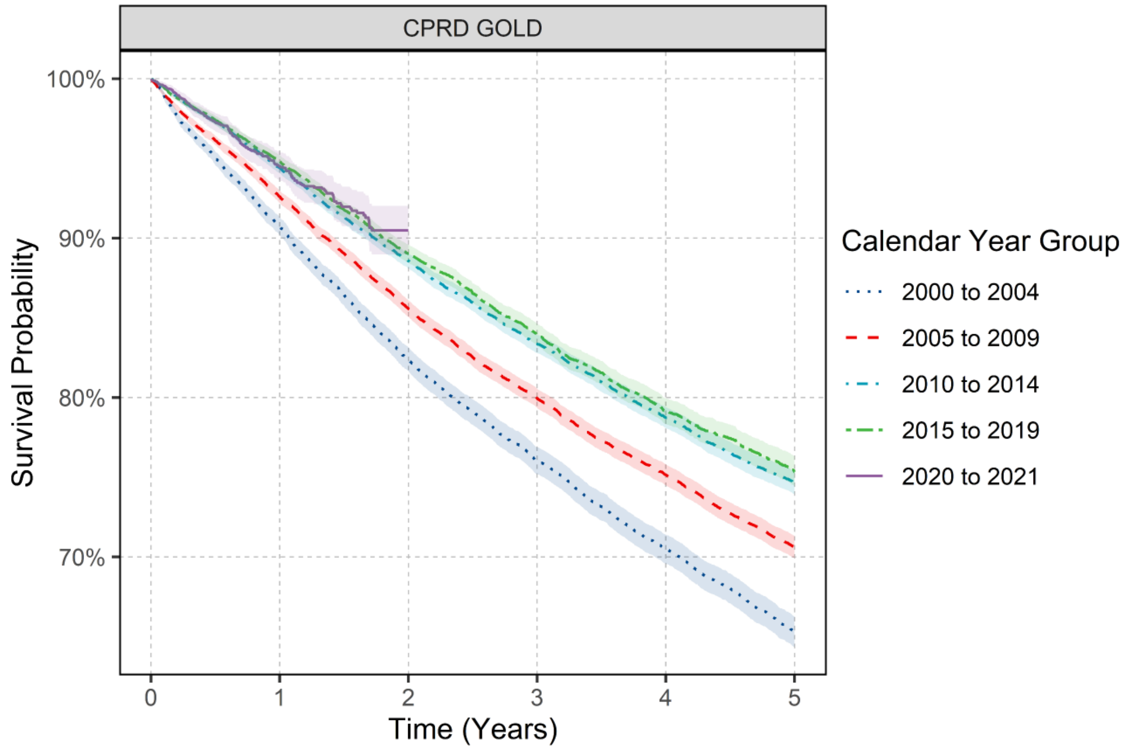

eTable 9: Survival After 1 and 5 Years Stratified by Calendar Year in CPRD GOLD

Time (year)	Calendar Year	% Survival (95% CI)
1	2000 to 2004	90.77 (90.21 to 91.34)
	2005 to 2009	92.60 (92.22 to 92.99)
	2010 to 2014	94.43 (94.10 to 94.76)
	2015 to 2019	94.83 (94.45 to 95.21)
	2020 to 2021	94.52 (93.64 to 95.41)
5	2000 to 2004	65.30 (64.35 to 66.27)
	2005 to 2009	70.62 (69.93 to 71.33)
	2010 to 2014	74.67 (73.95 to 75.39)
	2015 to 2019	75.34 (74.41 to 76.27)

CI: confidence interval

For results with less than 5 patients where CI were not computed the results are not reported.

© 2024 Tan EH et al. *JAMA Network Open*.

eTable 10: Survival After 1 Year Stratified by Age Group and Calendar Year in CPRD GOLD

Age Group	Calendar Year	% Survival (95% CI)
40 to 49	2000 to 2004	91.62 (84.08 to 99.83)
	2005 to 2009	98.00 (95.29 to 100.00)
	2010 to 2014	97.66 (95.41 to 99.95)
	2015 to 2019	97.73 (94.66 to 100.00)
	2020 to 2021	91.67 (77.29 to 100.00)
50 to 59	2000 to 2004	96.30 (94.98 to 97.63)
	2005 to 2009	97.98 (97.29 to 98.67)
	2010 to 2014	98.25 (97.62 to 98.88)
	2015 to 2019	98.77 (98.17 to 99.37)
	2020 to 2021	100.00 (100.00 to 100.00)
60 to 69	2000 to 2004	95.84 (95.10 to 96.59)
	2005 to 2009	97.17 (96.74 to 97.61)
	2010 to 2014	97.51 (97.11 to 97.90)
	2015 to 2019	98.16 (97.75 to 98.58)
	2020 to 2021	97.22 (96.06 to 98.40)
70 to 79	2000 to 2004	92.03 (91.21 to 92.86)
	2005 to 2009	93.05 (92.44 to 93.66)
	2010 to 2014	95.23 (94.73 to 95.74)
	2015 to 2019	95.64 (95.07 to 96.20)
	2020 to 2021	95.41 (94.16 to 96.67)
80 to 89	2000 to 2004	82.42 (80.84 to 84.03)
	2005 to 2009	84.44 (83.24 to 85.66)
	2010 to 2014	86.97 (85.80 to 88.14)
	2015 to 2019	86.84 (85.38 to 88.33)
	2020 to 2021	87.20 (83.88 to 90.65)
90+	2000 to 2004	69.46 (63.91 to 75.48)
	2005 to 2009	68.26 (63.73 to 73.12)
	2010 to 2014	75.18 (71.02 to 79.58)
	2015 to 2019	68.69 (63.27 to 74.57)
	2020 to 2021	72.65 (63.07 to 83.68)

CI: confidence interval

For results with less than 5 patients where CI were not computed the results are not reported.

© 2024 Tan EH et al. *JAMA Network Open*.

eTable 11: Survival After 5 Years Stratified by Age Group, and Calendar Year in CPRD GOLD

Age Group	Calendar Year	% Survival (95% CI)
40 to 49	2000 to 2004	70.98 (58.78 to 85.71)
	2005 to 2009	90.37 (84.57 to 96.57)
	2010 to 2014	92.68 (88.55 to 97.01)
	2015 to 2019	94.60 (89.46 to 100.00)
50 to 59	2000 to 2004	84.65 (82.09 to 87.29)
	2005 to 2009	89.47 (87.91 to 91.05)
	2010 to 2014	90.66 (89.10 to 92.25)
	2015 to 2019	91.63 (89.67 to 93.64)
60 to 69	2000 to 2004	80.96 (79.46 to 82.48)
	2005 to 2009	84.86 (83.88 to 85.86)
	2010 to 2014	85.99 (85.01 to 86.98)
	2015 to 2019	86.54 (85.22 to 87.88)
70 to 79	2000 to 2004	66.04 (64.57 to 67.54)
	2005 to 2009	70.24 (69.11 to 71.39)
	2010 to 2014	74.83 (73.67 to 76.02)
	2015 to 2019	76.27 (74.82 to 77.75)
80 to 89	2000 to 2004	41.81 (39.68 to 44.05)
	2005 to 2009	43.00 (41.24 to 44.82)
	2010 to 2014	48.20 (46.18 to 50.31)
	2015 to 2019	45.78 (43.05 to 48.69)
90+	2000 to 2004	14.11 (10.06 to 19.79)
	2005 to 2009	22.29 (17.95 to 27.67)
	2010 to 2014	22.77 (18.08 to 28.67)
	2015 to 2019	15.62 (10.17 to 23.99)

CI: confidence interval

For results with less than 5 patients where CI were not computed the results are not reported.

© 2024 Tan EH et al. *JAMA Network Open*.
