# Supplementary material for: Incidence, Prevalence, and Survival of Prostate Cancer in the UK
Source: JAMA Netw Open. 2024 Sep 19;7(9):e2434622. doi: 10.1001/jamanetworkopen.2024.34622 (PMC12315704; doi:10.1001/jamanetworkopen.2024.34622)
Supplement: Supplement 2. — Data Sharing Statement [file jamanetwopen-e2434622-s002.pdf]

Data Sharing Statement

Tan. Incidence, Prevalence, and Survival of Prostate Cancer in the UK. *JAMA Netw Open*. Published September 19, 2024. doi:10.1001/jamanetworkopen.2024.34622

Data
Data available: No

Additional Information
Explanation for why data not available: This study is based in part on data from the Clinical Practice Research Datalink (CPRD) obtained under licence from the UK Medicines and Healthcare products Regulatory Agency. The data is provided by patients and collected by the NHS as part of their care and support. The interpretation and conclusions contained in this study are those of the author/s alone. Patient level data used in this study was obtained through an approved application to the CPRD (protocol number 22_001843) and is only available following an approval process to safeguard the confidentiality of patient data. Details on how to apply for data access can be found at https://cprd.com/data-access.
